# Dosimetric characteristics of a cubic‐block‐piled compensator for intensity‐modulated radiation therapy in the Pinnacle radiotherapy treatment planning system

**DOI:** 10.1120/jacmp.v8i1.2323

**Published:** 2007-02-28

**Authors:** Koji Sasaki, Yasunori Obata

**Affiliations:** ^1^ Department of Radiological and Medical Sciences Nagoya University Graduate School of Medicine Nagoya Japan; ^2^ School of Radiological Technology Gumma Prefectural College of Health Sciences Gumma Japan; ^3^ Department of Health Sciences Nagoya University Graduate School of Medicine Nagoya Japan

**Keywords:** Intensity‐modulated radiation therapy, compensator, effective attenuation coefficient, physical modulator

## Abstract

We examined the dose distributions generated by ${\text{Pinnacle}}^{3}$ (Philips Radiation Oncology Systems, Milpitas, CA) for intensity‐modulated radiotherapy (IMRT) plans using a cubic‐block‐piled compensator as the intensity modulator for 4‐MV and 10‐MV photon beams. The Pinnacle treatment planning system (TPS) uses an algorithm in which only the physical density of the absorber is required for calculating the characteristics of the modulator. The intensity modulator consists of cubic blocks (attenuator) of a tungsten alloy, plus cubic blocks of polyethylene resin foam that fill the spaces between the attenuator blocks and polymethyl methacrylate (PMMA) boards that act as the platform for the modulator. By measuring the transmission for various thicknesses of attenuator and by deriving values for the total physical density of the modulator, we determined the optimal effective density by comparing the curves fitted for the actual transmission data with the transmission calculated by the TPS. Using these effective densities, we examined the accuracy of ${\text{Pinnacle}}^{3}$ for dose profiles of specific geometric patterns. The levels of consistency between the measurements and the calculations were within a tolerance of 3% of the dose difference and had a 3‐mm distance to agreement for the ladder‐, stairstep‐, and pyramid‐shaped test patterns, except in the high dose gradient region. In this modulator assembly, leakage occurred from the slits between the cubic blocks. This leakage was about 1.6% at maximum, and its influence on dose distribution was not crucial. In the TPS, in which physical density was the only user‐controllable parameter, we used the effective density of the absorber deduced from the effective mass attenuation coefficient. We conclude that the intensity modulation compensator system, together with a piled cubic attenuator, is clinically applicable, with an acceptable tolerance level. For the intensity map of the IMRT plan, measurements in treatment fields met 3% and 3‐mm criteria, excluding some regions of high gradient, which had a discrepancy of less than 5% and 4 mm.

PACS numbers: 87.53.Mr, 87.53.Tf

## I. INTRODUCTION

The aim of radiation therapy is to deliver an adequate dose of radiation to a tumor while limiting exposure to the surrounding normal tissue so as to minimize the risk of radiation injury. In conventional radiation therapy, the intensity of the administered radiation dose is uniform within each irradiation field. However, improved three‐dimensional (3D) radiation therapy planning systems have allowed the radiation dose to be focused onto the tumor volume in intensity‐modulated radiation therapy (IMRT).

A common method for modifying the intensity of radiation within a field of irradiation involves the use of a multileaf collimator (MLC).^(1)^ This method has attracted controversy because of difficulties linked to control over the position and movement precision of the MLC and to dosimetric accuracy of the linear accelerator in delivering small monitor units (MUs).(^2^–^9^) In addition, the prolonged treatment time associated with the increased number of MUs in segmental MLC IMRT has been acknowledged to be a problem.(^1^,^10^–^13^)

On the other hand, when compensators are used to modulate dose intensity within a radiation field, a quality control for mechanical precision and dosimetric accuracy is easy because the field size is fixed. Thus, IMRT technique is believed to be achievable with a conventional linear accelerator that is not equipped with a MLC. Reported methods that involve the use of compensators for IMRT include one in which the compensator shape is formed using a milling machine(^5^,^14^–^17^) and another that involves the piling of cubic blocks for compensation.(^18^–^20^)

In the present study, we optimized the input parameter for a commercial treatment planning system [the TPS for ${\text{Pinnacle}}^{3}$ (Philips Radiation Oncology Systems, Milpitas, CA)] that was used for IMRT planning with a cubic‐block‐piled compensator, and we examined the extent of leakage from the slits and pinholes in the cubic block assembly.

Compared with the MLC IMRT and the milling machine–generated compensator, the cubic‐block‐piled compensator IMRT has a limitation related to the resolution of radiation intensity. For example, because the size of a cubic block is 0.5×0.5×0.5 cm, the intensity resolution that can be achieved is only about 0.9 cm on an isocenter plane. In addition, the intensity modulation range achievable by one of the blocks is approximately 27% of the open field for 4‐MV photons and 22% for 10‐MV photons. These limitations need to be taken into consideration for clinical use. Moreover, with this system, the IMRT is adequate only for cases in which the maximum field size is smaller than 9×9 cm.

In our clinic, IMRT using the compensator is planned on the ${\text{Pinnacle}}^{3}$ TPS. To calculate compensator thickness, the TPS requires input of the physical density of the cubic block. We therefore used measurements of depth dose to evaluate an optimal effective density of the cubic block. Based on the results, we evaluated the suitability of the physical modulator system for clinical use.

## II. MATERIALS AND METHODS

In the present study, we used photon beams of 4 MV and 10 MV generated by a medical linear accelerator system (ML‐20MDX: Mitsubishi Electric, Tokyo, Japan), and we employed these phantoms for radiation dosimetry:

- A 3D scanning water phantom system (RFA300: Scanditronix–Wellhofer, Nuremburg, Germany)
- A one‐dimensional (1D) water phantom (WP‐1D: Scanditronix–Wellhofer)
- A water‐equivalent solid phantom (Tough Water: Kyoto Kagaku, Kyoto, Japan)

The measurement devices included a 0.12‐cm^3^ thimble ionization chamber (RK: Scanditronix–Wellhofer), a 0.6‐cm^3^ thimble ionization chamber (30013: PTW, Freiburg, Germany), and a 0.015‐cm^3^ thimble ionization chamber (31014: PTW), which were connected to an electrometer (Ramtec 1000 Plus: Toyo Medic, Tokyo, Japan). Furthermore, we used radiographic film (EDR2: Eastman Kodak Company, Rochester, NY) for some measurements. To analyze dose distribution with film, we used a 16‐bit, 150‐dpi, single‐channel, twain‐interface, flatbed film scanner (ES‐10000G: Epson, Tokyo, Japan), and dose comparison software (DD System: R‐TEC, Tokyo, Japan). The DD System forces the scanner to self‐calibrate before scanning a film, and it supports optical density ranging from 0.07 to 3.80. The calibration enables conversion of the scanner's digital readout value to optical density. By default, a 3×3 median filter was used to filter the scanned films. For IMRT planning, we used the ${\text{Pinnacle}}^{3}$ TPS, version 7.4f. A regular compensator program incorporated into the TPS was used. In ${\text{Pinnacle}}^{3}$, primary beam attenuation, primary beam hardening, the scatter from the modifier, and the phantom scatter are taken into consideration in the computation of the dose at a point in the medium.

**Figure 1:**
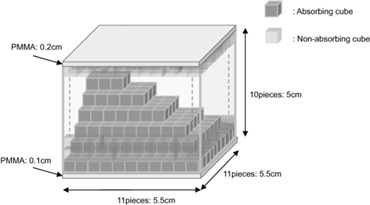
Schematic illustration of the inside of the compensating filter assembly. The dark‐gray cubes are the absorbing material (tungsten alloy) and the pale‐gray cubes are the non‐absorbing material (polyethylene resin foam). Polymethyl methacrylate (PMMA) boards were placed on the top and bottom of the assembly for the modulator platform. Each cube measures 0.5×0.5×0.5 cm.

**Figure 2:**
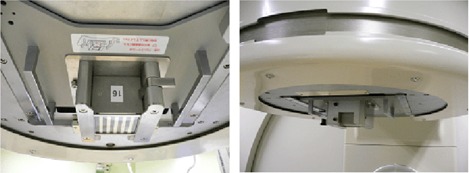
Photographs of the cubic‐block‐piled compensator mounted on the accessory mount of the linear accelerator gantry head.

As an absorbing device for beam modulation, we used a physical modulator (TETRIS‐RT: Apex Medical, Tokyo, Japan).^(20)^ The absorbing cubic blocks consist of a mixture of 96% tungsten and 4% polycaprolactam amide, with a physical density of $12\;g/{\text{cm}}^{3}$. The non‐absorbing cubic blocks used to fill the spaces between the absorbing cubic blocks consist of 100% polyethylene resin foam of $0.094\;g/{\text{cm}}^{3}$ physical density, with each piece being 0.5×0.5×0.5 cm in size. Fig. 1 is a schematic illustration of the inside of the compensating filter device. The photographs in Fig. 2 show the cubic‐block‐piled compensator mounted on the accessory mount of the linear accelerator gantry head. The cubic blocks were stacked to a maximum of 11×11×10 pieces (5.5×5.5×5 cm) and placed inside a stainless steel enclosure. The enclosure contains a polymethyl methacrylate (PMMA) board of 1 mm thickness at the bottom and a second PMMA board of 2 mm thickness at the top. These boards are used to retain the absorbers at the point at which the beams pass through. The distance from the source to the bottom of the cubic block assembly is 60 cm, with an available maximum irradiation field of 9.16×9.16 cm at the isocenter plane. However, in this study, the irradiation field through the upper and lower collimators was fixed at 9×9 cm.

First, an IMRT plan with ideal dose distribution was planned by an optimization program for conventional IMRT. That plan was then converted into a matrix for the compensator. A pattern for the compensator, copied from a treatment port of the IMRT plan, consisted of 10 layers in the thickness direction. One layer of the cubic block pattern was made automatically by the arrangement machine, and the pattern was inspected by the sensor system. That process was repeated in each layer to complete the total cubic pattern. The compensator was constructed in about 8 minutes.

### A. Attenuation characteristics of the absorbing cubic blocks, non‐absorbing cubic blocks, and retention boards

The physical modulator that we used has a composite configuration in which non‐absorbing cubic blocks fill the spaces between the absorbing cubic blocks. In addition, the retention system contains PMMA boards. It was necessary to identify the attenuation characteristics of the individual materials relative to the photons emitted by the linear accelerator. We measured the attenuation characteristics of the 4‐MV and 10‐MV photon beams relative to the absorbing and the non‐absorbing cubic blocks and to the PMMA boards. For those measurements, we used narrow beams with an irradiation field of 3.2×3.2 cm at the isocenter plane. We attached a proper build‐up cap to the 0.6‐cm^3^ thimble ionization chamber and measured the relative doses at the source‐to‐chamber distance of 2 m in air.

### B. Depth‐dose curve changes and influence on the penumbra region of the radiation field with compensator

Modulating the primary beams with high‐density absorbers such as those made of tungsten can cause beam hardening, which results in changes in the depth‐dose characteristic. We therefore measured the depth dose when cubic blocks were inserted in varying thicknesses into the 4‐MV and 10‐MV photon beams.

The effect of the compensator on dose distribution in the penumbra region was evaluated by comparing the dose gradient in open and compensation fields on a crossplane at a 10‐cm depth. We used the 3D scanning water phantom system and the 0.12‐cm^3^ thimble ionization chamber at a source‐to‐surface distance of 90 cm and a depth of 10 cm. A scan pitch of 2 mm was used.

Dose profile, $D_{i}^{\prime }$, at the *i*th point on the cross‐plane is defined by the equation

(1) $$D_{i}^{\prime }=\frac{D_{i}-D_{\text{axis}}}{D_{\text{axis}}}\times 100,$$

where $D_{i}$ is the dose at *i*th point and $D_{\text{axis}}$ is the dose on the central axis.

The dose gradient, $dD_{i}$, at *i*th point is then defined by

(2) $$dD_{i}=\frac{D_{i-1}^{\prime }-D_{i+1}^{\prime }}{\Delta },$$

where Δ is the distance between the (i−1)th and (i+1)th points.

**Figure 3:**
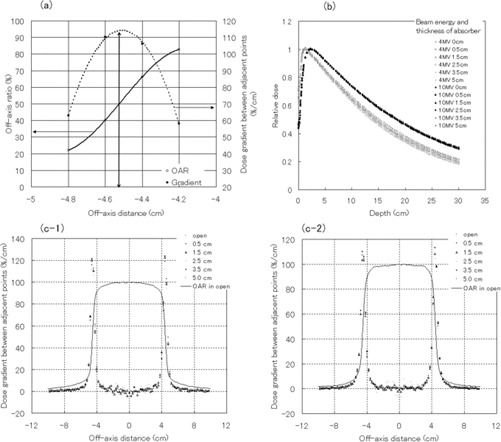
(a) Relations with off‐axis ratio curve and rate of change of relative dose between adjacent points. (b) Depth‐dose curves measured in water for various absorber thicknesses. The curves are normalized for $d_{\max}$. The open symbols indicate the depth‐dose of 4‐MV photons. The closed symbols indicate the depth‐dose of 10‐MV photons. (c) Dose gradient between adjacent points (percent/centimeter) for various thickness of absorber at a depth of 10 cm in water. The dose profile curve of the open field is superimposed on the same figure. (c−1) Curves for 4‐MV photons. (c−2) Curves for 10‐MV photons.

A curve of dose profile proximal to the 50% dose point is generally convex on the axis side, but concave on the edge side. Therefore, a curve of dose gradient has an inflexion point. Fig. 3(a) illustrates the off‐axis ratio and the rate of change of the dose proximal to the 50% dose point for the central‐axis dose. The point of 50% relative dose can be determined from the local maximum of the rate‐of‐change curve. Using that approach, we determined the change in the pattern of penumbra region when cubic blocks were inserted into the field in varying thicknesses.

### C. Leakage from the slits between the absorbing cubic blocks

The modulator assembly has the potential to generate pinholes and slits between the piled absorbing cubic blocks. Each corner of an individual cubic block is rounded with a radius of 0.2 mm during the production process, allowing for the formation of pinholes. It was therefore necessary to examine the influence of leakage from the inter‐block slits and pinholes. We used extended dose range film to assess the leakage. The film was placed perpendicular to the central axis of the beams at a depth of 10 cm in the 30‐cm‐thick solid‐water phantom. The thickness of the absorber stack was varied six levels from 0 cm to 5 cm. In open fields, the MUs were set to 100, and in blocked fields, preset MUs from 300 to 900 were used.

To yield relations of a dose and optical density, we carried out measurements as follows. The film was placed perpendicular to the beam axis at a depth of 10 cm in the solid‐water phantom. Up to 300 MUs and a 10×10 cm field size were used. Each set of sensitometric and profile film exposures used the same batch of film and were developed at the same time. Before any measurement, the system was calibrated using an absolute dose measured by ionization chamber. At the time of the film dosimetry, a related curve of optical density and dose was used to convert measured optical density to absorbed dose. A similar film calibration procedure was used for all of the tests described in the subsections that follow.

### D. Changes in the effective linear attenuation coefficient through absorbers in water

It is generally accepted that, for a given photon energy, changes in radiation field size and depth affect the effective linear attenuation coefficient. In the present study, the irradiation field was fixed at 9×9 cm, and so the size of the irradiation field did not need to be taken into consideration. To identify the beam characteristics affected by depth variation, we varied the absorber thickness and measured the resulting transmission ratio on the central axis of the beams at depths of 5 cm, 10 cm, and 15 cm in water. For these measurements, the 1D water phantom and 0.6‐cm^3^ thimble ionization chamber were used.

### E. Determination of cubic block density

The Pinnacle TPS provides an attenuation coefficient based on input giving the densities of the absorbers. Absorber density and thickness are the only parameters that the user can change.

We placed the 0.6‐cm^3^ thimble ionization chamber on the central axis of the beam at a depth of 10 cm in water and measured the absolute absorbed dose. We then compared the measured dose with the calculated dose obtained for various absorber densities in the TPS.

Exponential attenuation is the basis of the photon beam attenuation measurement. The attenuation that relates to the monochromatic energy spectrum of narrow beams can be expressed with the equation

(3) $$I=I_{0}\;\exp(-\mu ∙t),$$

where *I* is the intensity of radiation transmitted through an attenuator of thickness *t* inserted into the beam, and μ is the linear attenuation coefficient for the attenuator material.

However, because our modulator system includes three types of material, Equation 3 is modified to become

(4) $$I=I_{0}\;\exp[-(\mu_{1}t_{1}+\mu_{2}t_{2}+\mu_{3}t_{3})],$$

where $\mu_{1},\mu_{2},\;\text{and}\;\mu_{3}$, represent the linear attenuation coefficients, and $t_{1},t_{2},\;\text{and}\;t_{3}$ represent the thicknesses of, respectively, the absorbing cubic blocks, the non‐absorbing cubic blocks, and the PMMA boards.

In addition, the equation that follows holds true, because the total thickness *T* of the absorbing and non‐absorbing cubic blocks is always 5 cm:

(5) $$T=t_{1}+t_{2}=5.$$

Furthermore, $t_{3}$ is always a constant. Therefore, Equation 4 is transformed by Equation 5 to give

(6) $$\ln\frac{I}{I_{0}}=-(\mu_{1}-\mu_{2})t_{1}-(5\mu_{2}+\mu_{3}t_{3}).$$

The TPS demands only the density of the high‐atomic‐number absorbers; the densities of the filling materials are not required. Density ρ is defined as the effective density for the whole compensation system, and the second term on the right side of Equation 6 is constant. When that term is replaced with constant C,

(7) $$C=-(5\mu_{2}+\mu_{3}t_{3}),$$

Equation 6 is then rearranged to yield the expression

(8) $$\ln\frac{I}{I_{0}}=-\frac{\left(\mu_{1}-\mu_{2}\right)}{\rho_{\text{eff}}}t_{1}\rho_{\text{eff}}+C,$$

where $\left[(\mu_{1}-\mu_{2})/\rho_{\text{eff}}\right]$ represents the effective mass attenuation coefficient and $\rho_{\text{eff}}$ is thus the effective density of the absorbing material.

### F. Dose verification of geometric patterns

To confirm the accuracy of the cubic‐block density obtained by the procedures described in subsection E, we determined the dose distributions for various geometric patterns in ${\text{Pinnacle}}^{3}$, and compared those distributions with a dose measured by pinpoint ionization chamber (0.015 cm^3^) and by EDR2 film. The verification patterns used the cubic configuration with ladder‐, stairstep‐, and pyramid‐shaped dose distributions. In the ladder pattern, the absence and presence of absorbers of maximum thickness 5 cm were alternated. In the stairstep pattern, the thickness of the absorbers was increased in 0.5‐cm steps from 0 (no absorbers) to a maximum of 5 cm. In the pyramid pattern, no absorber was placed in the center, and the thickness of the absorbers was increased from the center in 1‐cm steps to a maximum thickness of 5 cm at the sides.

The measurements were performed in water on an isocenter plane of 10 cm depth perpendicular to the beam axis. The measurements with the ionization chamber were performed in the 3D scanning water phantom at multiple points by an interval of *2*–4 mm. The film measurements were performed in the solid‐water phantom, and a few holes were pricked along the laser lines on the film envelope to set a position precisely.

For the dose calculation, we used the collapsed cones convolution superposition algorithm with a voxel size of 2×2×2 mm. The pixel size of the distribution display was 1×1 mm.

### G. Dose verification of a benchmark case

Using the benchmark test pattern suggested by the National Cancer Institute,(^21^) we generated a five‐portal IMRT irradiation plan with ${\text{Pinnacle}}^{3}$. We converted each port of the ideal plan into a 0.5‐cm matrix and converted the intensity resolution into 10 steps for modulator construction. Subsequently, we recalculated this plan on a water‐equivalent solid phantom and compared it with the dose distribution obtained by film dosimetry on two transverse slices of the composite plan. Additionally, a comparison of the dose distributions from the calculated and measured doses was performed on each single field in the same way that the composite distribution was compared. However, this time, the individual beam was incident on the surface of the solid‐water phantom perpendicularly. We used the earlier described DD System to perform the comparisons and analyses of the dose distributions.

The dose verification in the solid‐water equivalent phantom was performed by using the 0.6 cm^3^ ionization chamber for two points in the target volume. The measured points were the isocenter and a point of low dose‐gradient (that is, a point 1.7 cm right and 0.5 cm back from the isocenter). The chamber reading was compared with the calculated mean dose over the chamber volume.

## III. RESULTS

### A. Attenuation characteristics of the absorbing cubic blocks, non‐absorbing cubic blocks, and retention boards

The half‐value layer of the absorbing cubic blocks was 1.23 cm with 4‐MV photon irradiation and 1.38 cm with 10‐MV photon irradiation. The measured transmission through the target absorbers with maximum total thickness of 5 cm was 6.35% for 4‐MV photons and 8.27% for 10‐MV photons. The corresponding values for 5 cm of non‐absorbing cubic blocks and PMMA boards was 94.72% for 4‐MV photons and 96.24% for 10‐MV photons. The PMMA boards accounted for 2.22% of the attenuation at 4 MV and for 1.67% at 10 MV.

### B. Depth‐dose curve changes and influence on a penumbra region of radiation field with compensator

Fig. 3(b) shows the changes in the depth‐dose curve in water for various thicknesses of absorber. The curves are normalized for the dose measured at the depth of maximum dose. With variations in absorber thickness, the depth‐dose curve showed only small changes at 10 MV. However, at 4 MV, the slopes of the curves at positions deeper than the depth of the maximum dose decreased because of the beam hardening effect with the increased thickness of absorber. When a tungsten absorber thickness of 5 cm was used, the difference in the relative dose at a depth of 15 cm for 4‐MV photons was 5.1%, and for 10‐MV photons, it was 0.9%.

Fig. 3(c‐1,c‐2) represent the dose profile and the dose gradient of a relative dose between adjacent points. The dose gradient at points in the penumbra region did not depend on the thickness of the cubic blocks at either energy setting [Fig. 3(c)]. In addition, a point of 50% dose was constant regardless of the thickness of the cubic blocks and open beam. The point with the greatest rate of change was a point ±4.5 cm out of axis. For these reasons, we conclude that the shape of the dose profile in the penumbra region does not vary with compensator thickness.

### C. Leakage from the slits between the absorbing cubic blocks

Film dosimetry showed leakage from the inter‐block slits and pinholes of the absorbing cubic blocks. The extent of the leakage decreased with increasing distance from the central axis of the beams. Table 1 shows the maximum values of the leakage relative to the dose of the central beam axis. The ratios of the leakage from the slits were within approximately 1.6%, and these ratios were approximately constant and showed no dependence on the thickness of the absorber. In addition, the maximum ratio of the leakage for the pinholes increased as the total thickness of the absorbers increased. For pinholes, the increment of the leakage versus absorber thickness was proportional to transmission on the central axis.

**Table 1:** The leakage from slits and pinholes between the cubic blocks of the absorber

	Maximum leakage at 10‐cm depth (%)			
	From slit		From pinhole	
Thickness of absorber (cm)	4 MV	10 MV	4 MV	10 MV
0.5	1.1	1.1	1.3	1.2
1.5	1.6	1.4	2.9	2.5
2.5	1.6	1.6	5.8	3.4
3.5	1.1	1.5	6.9	4.1
5.0	1.3	1.5	8.3	7.6

### D. Changes in the effective linear attenuation coefficient through absorbers in water

**Figure 4:**
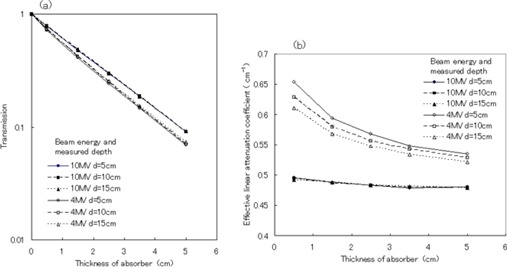
(a) Transmission curves as a function of absorber thickness at depths of 5 cm, 10 cm, and 15 cm in water. (b) Comparison of effective linear attenuation coefficients as a function of absorber thickness at depths of 5 cm, 10 cm, and 15 cm in water. The open symbols indicate the curves for 4‐MV photons. The closed symbols indicate the curves for 10‐MV photons.

Fig. 4(a,b) shows changes in the transmission ratio and effective linear attenuation coefficient as a function of absorber thickness at depths of 5 cm, 10 cm, and 15 cm in water. With 4‐MV photons, the differences in the transmission ratios at depths of 5 cm and 15 cm, normalized for the transmission ratio at a depth of 10 cm, were −3.0% and 3.8% respectively [Fig. 4(a)]. However, the transmission ratio for 10‐MV photons was only slightly affected by depth.

Fig. 4(b) shows that the change in the effective linear attenuation coefficient for 10‐MV photons was only 0.67%, regardless of depth changes. However, the coefficient for 4‐MV photons displayed a maximum change of 6.47% and depended on both absorber thickness and depth in water.

### E. Determination of the density of cubic blocks

**Figure 5:**
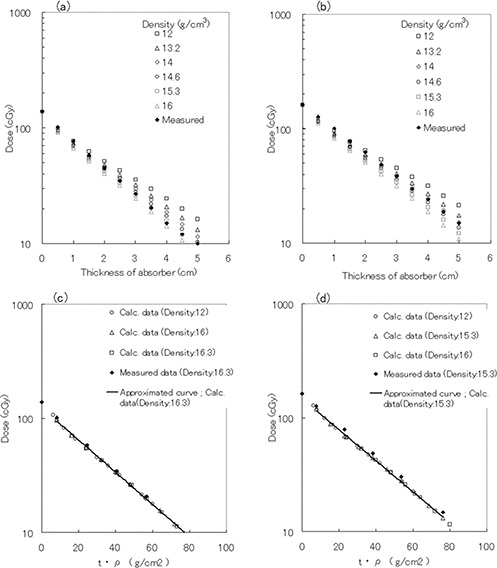
Transmission curves calculated by the treatment planning system using various absorber density values (open symbols) and the measured doses at a depth of 10 cm in water (closed symbols). (a) Transmission curve for 4‐MV photons as a function of absorber thickness. (b) Transmission curve for 10‐MV photons as a function of absorber thickness. (c) Transmission curve for 4‐MV photons as a function of thickness density *t*ρ. (d) Transmission curve for 10‐MV photons as a function of thickness density *t*ρ. t=the thickness of the absorber; ρ=the density of the absorber.

Fig. 5(a,b) shows the transmitted dose obtained by entering various absorber density values into the TPS (dose measured at 10 cm depth in water). The only variable permitted by the TPS is the physical density of the absorber. The calculation by the TPS therefore models radiation transmission through an absorber using a mass attenuation coefficient.

After the thickness of the absorber, *t*, was transformed into *t*ρ, the transmission data were plotted for 4‐MV photons [Fig. 5(c)] and for 10‐MV photons [Fig. 5(d)]. In the resulting graphs, the slopes of the transmission curves are (μ/ρ)s, and we estimated the values of the slopes from the fitting lines. The effective density $\rho_{\text{eff}}$ was obtained from Equation 8 using the known values of $\left[(\mu_{1}-\mu_{2})/\rho \right]$ and thickness *t*. Thus, the effective density of the absorber was $16.3\;g/{\text{cm}}^{3}$ for 4‐MV photons and $15.3\;g/{\text{cm}}^{3}$ for 10‐MV photons. The determination coefficients of the approximate curves were 0.9994 and 0.9998 respectively.

### F. Dose verification of geometric patterns

**Figure 6:**
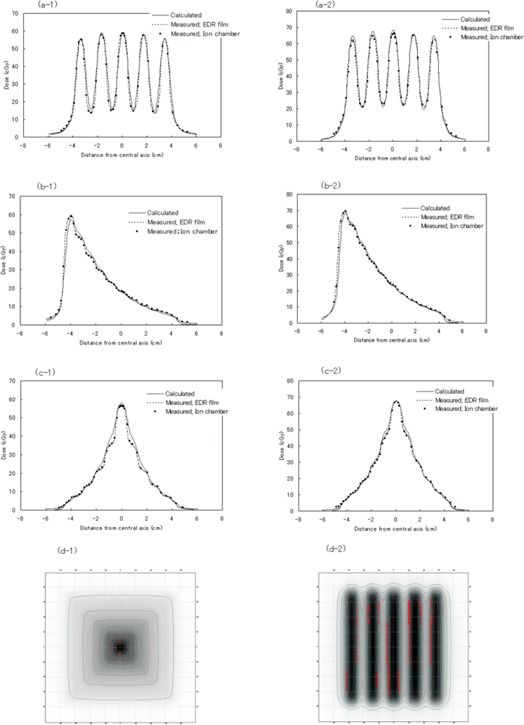
Comparison between the measured dose and calculated dose after optimization of absorber density. The solid line is the dose profile calculated by the treatment planning system. The dashed line is the dose profile measured using EDR2 film. Closed circles indicate the dose measured by the pinpoint chamber. The beam energy was 4 MV for the graphs on the left and 10 MV for the graphs on the right. (a) Ladder pattern. (b) Stairstep pattern. (c) Pyramid pattern. (d) The gamma‐value map. Map at left is over pyramid pattern; map at right is over ladder pattern. The areas showing a red color are those that exceed the 3% and 3 mm criteria.

Fig. 6 shows the comparison between the calculated doses and the measured doses following optimization of absorber density and also the output parameters based on the results described in the previous subsections. According to the gamma dose distribution comparison method,^(22)^ which is a combined evaluation of dosimetric and spatial deviations, almost the entire segment of all patterns satisfied a tolerance of 3% dose difference and 3‐mm distance to agreement. However, in the portion with a high dose gradient, the differences were within 5% and 4‐mm criteria.

Fig. 6(d) shows the gamma‐value map for two patterns that have high dose gradient areas. The portions shown in red exceed the 3% and 3‐mm criteria. In those areas, the TPS overestimates the gradient of the dose steps. On the other hand, the difference between the film measurement and the ionization chamber measurement is less than 3% within a field on all patterns.

### G. Dose verification of a benchmark case

**Figure 7:**
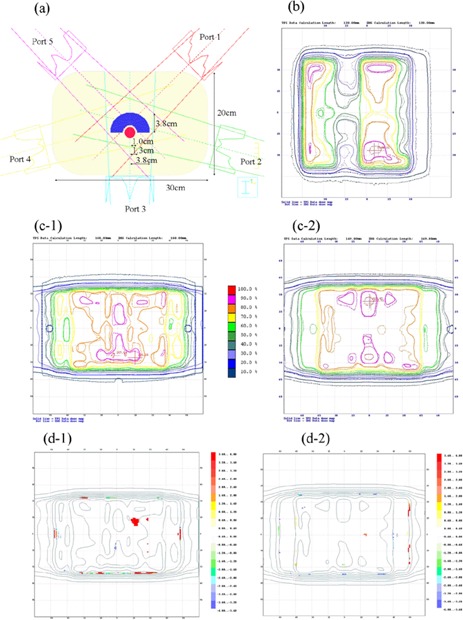
(a) Schematic image of the five‐portal irradiation test using a concave‐shaped target volume (blue) and a tubular volume for avoidance (red). (b) Comparison between the measured and calculated values in a single portal from the 315‐degree direction for 10‐MV photons. (c) Comparison between the measured and calculated values in the coronal plane for five‐portal IMRT. (c‐1) 4‐MV photons. (c‐2) 10‐MV photons. (d) The gamma distribution over the coronal planes for the 4% and 3‐mm criteria. (e‐1) 4‐MV photons. (e‐2) 10‐MV photons. The solid line for Fig. 7(b,c,d) is the dose distribution calculated by the treatment planning system. The dashed line is the dose distribution measured by EDR2 film (Eastman Kodak Company, Rochester, NY).

Fig. 7 shows the comparison between the dose distributions obtained from the TPS and those obtained by film dosimetry. Fig. 7(b) shows the dose distribution for port 5 (315‐degree gantry angle) for the five‐portal irradiation using 10‐MV photons. It indicates a discrepancy within the 3% and 3‐mm criteria over the entire field. The discrepancies for the other four ports were also within 3%. The results with 4‐MV photons were similar.

Fig. 7(c,d) shows the dose distribution and gamma distribution over the coronal planes, for which the discrepancy in the irradiated region was within approximately 4% and 3‐mm criteria.

**Figure 8:**
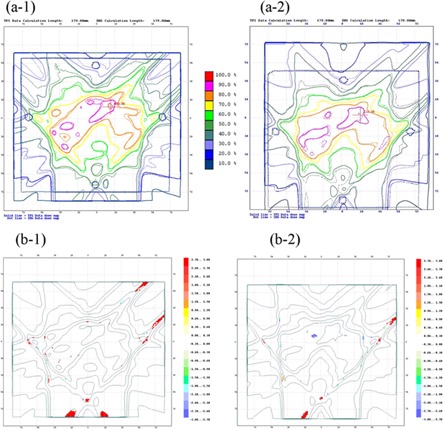
(a) Comparison between the measured value and the calculated value in the axial plane for five‐portal intensity modulated radiotherapy. The solid line is the dose distribution calculated by the treatment planning system. The dashed line is the dose distribution measured by EDR2 film (Eastman Kodak Company, Rochester, NY). (a‐1) 4‐MV photons. (a‐2) 10‐MV photons. (b) The gamma distribution over the axial planes for 3% and 3‐mm criteria. (b‐1) 4‐MV photons. (b‐2) 10‐MV photons.

Fig. 8(a,b) shows the dose distribution and gamma distribution over the axial planes, for which the discrepancy was approximately within the 3% and 3‐mm criteria.

Table 2 gives the ionization chamber measurements. The agreement of these ionization chamber measurements with the TPS was good; the largest difference was 2.79%.

**Table 2:** Ionization chamber measurements for verification of calculated dose

Energy	Position	Dose (cGy)		Difference
		Calculated	Measured	(%)
4MV^a^	Isocenter	194.9	195.1	0.10
	Low dose–gradient point^b^	210.0	204.3	−2.79
10MV^a^	Isocenter	198.4	199.3	0.45
	Low dose–gradient point^b^	189.8	189.5	−0.15
4MV^c^	Isocenter	191.4	187.3	−2.19
	Low dose–gradient point^b^	209.8	206.9	−1.40
10MV^c^	Isocenter	195.1	197.4	1.17
	Low dose–gradient point^b^	187.5	185.2	−1.24

a Measured by 0.6‐cm^3^ ionization chamber.

b Coordinate of right 1.7 cm and backward 0.5 cm from the isocenter.

c Measured by 0.015‐cm^3^ ionization chamber.

## IV. DISCUSSION

Currently, the compensator‐like intensity modulator is not in common use for IMRT delivery. However, a solid IMRT system of this type is one of the methods that deliver IMRT in accelerators without the MLC system.(^23^,^24^) Different compensator types have been used to improve dose distribution,(^14^–^20^,^25^,^26^) and we examined the possibility of applying IMRT with a cubic‐block‐piled compensator combined with ${\text{Pinnacle}}^{3}$.

The advantage of IMRT with compensators is the dose delivery method, which simplifies quality assurance. In our method using a cubic‐block‐piled compensator, intensity modulation is a discrete value, and production of the compensator is extremely easy. We have found that using a high‐density tungsten alloy yields a wide range of intensity modulation with both 4‐MV and 10‐MV photon beams.

When the compensator is inserted into the radiation field, the influence—such as a broadening effect on the penumbra region—may be given by the side scattering. However, when cubic blocks were inserted at thicknesses from 0.5 cm to 5.0 cm in a whole radiation field of 9×9 cm, a change in the penumbra region was not found as compared with open field.

Radiation leakage attributable to rounding of the cubic block edges was expected. As the thickness of the cubic‐block arrangement increased, variation of the dose in the dose profile curve was understood to be increased [Fig. 3(c)]. Leakage from the inter‐block slits of the cubic block assembly was approximately constant, at 1.6% of maximum leakage. It was almost same or lower than the inter‐leaf leakage of the MLC (2.5% of maximum leakage).^(4)^ In addition, the maximum ratio of the leakage from the pinholes relative to the central axis dose increased as the total thickness of the absorbers increased. This finding was attributable to a lack of important change in the leakage radiation for the pinholes and a decrease in transmission on the central axis as absorber thickness increased. It seems that the influence of the pinholes is not so important, because that influence is limited to a small area of full width at half‐maximum (2 mm). For evaluation of the five‐portal irradiation test, the measured dose variation at the slit and pinhole regions was not detectable. The extent of leakage decreased at off‐axis points because the nondivergent nature of the cubic blocks ensured that, for all off‐axis points, the chance of a direct divergent beam hitting the patient through pinholes was minimal.

The lower energy component of photon beams is known to be selectively removed by the compensator. In the present study, for the 4‐MV photon beam, the slopes of the depth‐dose curves changed with absorber thickness [Fig. 3(b)]. This beam‐hardening effect was weaker for 10‐MV photon beams (Fig. 4).

When an absorber is used in IMRT, various problems occur, such as dependence of the linear attenuation coefficient on depth and field size. In particular, because the depth‐dose characteristic used for the TPS is determined solely by the density of the absorber, the mentioned dependences are difficult to remove from depth‐dose calculations. We solved the problem of how to determine the effective density of an absorber deduced from the mass attenuation coefficient at 10 cm depth in water. The values for the effective densities were $16.3\;g/{\text{cm}}^{3}$ for a 4‐MV photon beam and $15.3\;g/{\text{cm}}^{3}$ for a 10‐MV photon beam (Fig. 5). Using those densities, adequate levels of consistency (3% and 3‐mm criteria) were noted between the calculated and the measured doses for the various test patterns. However, in the portion with a steep dose gradient, the differences were within 5% and 4‐mm criteria. The shapes in the measured dose distribution of a stairstep and a pyramid pattern appeared more clearly with 4‐MV photons than with 10‐MV photons (Fig. 6). Those variations depend on the difference in absorption coefficient. However, the variations were not reproduced in the adequately calculated dose distribution. These distinctions might be caused in the algorithm of the TPS by inadequate consideration of a change of scattered radiation. It would be one of the reasons that the beam modeling of the TPS is basically coordinated to reproduce the shape of an open field.

Using the five‐portal irradiation plan for a target volume with a concave shape and for a tubular‐shaped organ‐at‐risk volume in a phantom of cylindroid shape, we evaluated the planar dose distribution for each single field and for a multiple field of five ports. We performed the evaluation for each single port at a depth of 10 cm for the 4‐MV and 10‐MV beams, with the result that the agreement between the measured and calculated dose was within 3% and 3‐mm criteria over the entire field. Similarly, adequate levels (within 5% and 3‐mm criteria) were achieved in the evaluation of dose distributions from a total of five ports. Given that the depth dependence of the effective linear attenuation coefficient was smaller with 10‐MV photons than with 4‐MV photons, our prediction was that consistency between the calculated and measured doses with 10‐MV photons might be better than those with 4‐MV photons. But agreement in dose distribution did not differ between the two energies.

Our study showed several regions with a discrepancy above 3%, but overall agreement remained good. As will be understood, when this absorber is used clinically, in dose distribution for an individual patient, investigation of the agreement between planned and measured doses is necessary.

## V. CONCLUSION

We evaluated the efficacy of IMRT with a commercially available TPS (${\text{Pinnacle}}^{3}$), which uses the cubic‐block‐piled compensator as an intensity modulator for 4‐MV and 10‐MV photon beams.

This modulator system has some limitations related to

- maximum field size (9×9 cm),
- spatial resolution (0.9×0.9 cm) at the isocenter plane,
- practicable discrete dose levels (10),
- maximum transmission through tungsten blocks (6.35% for 4‐MV photons and 8.27% for 10 MV photons), and
- leakage from the slits between the absorbing tungsten cubic blocks (within 1.6%).

Nevertheless, in most regions, dose verification met 3% and 3‐mm criteria, and in all regions of target volume, it met 5% and 4‐mm criteria.

From the results, we conclude that, using an effective density (pseudo‐density), the multiple‐object compensator design in the ${\text{Pinnacle}}^{3}$ TPS allows for IMRT plans with clinically acceptable dosimetric accuracy. In addition, this intensity modulation compensator system with piled cubic blocks composed of tungsten alloy can be used clinically within an acceptable tolerance level with a linear accelerator that is not equipped with a high‐quality MLC.

## ACKNOWLEDGMENTS

We extend our deep gratitude to all personnel at the Treatment Technology Department of Fukuroi Municipal Hospital, who helped us to obtain the data for this study.

We are grateful to Dr. Masao Hoshina for his constructive comments during the preparation of the manuscript.
